# Code Not-So-Blue: Burden and Predictors of Non-urgent Visits to the Surgical Emergency Room in Peshawar, Pakistan

**DOI:** 10.7759/cureus.82973

**Published:** 2025-04-25

**Authors:** Muhammad Awais Khan, Abdul Haseeb, Fawad Ali, Elham Shakil, Ramisha Fatima, Uzma Wahid, Maseel Ahmad, Muhammad Moeed, Qaidar Alizai, Shehryar Khan

**Affiliations:** 1 Department of Trauma and Orthopaedic Surgery, Rehman Medical Institute, Peshawar, PAK; 2 Department of Surgery, Hayatabad Medical Complex Peshawar, Peshawar, PAK

**Keywords:** non-urgent, pakistan, peshawar, surgical emergency, triage

## Abstract

Background: Non-urgent visits to surgical emergency rooms (SERs) are an unnecessary burden on emergency health services, especially in low-resource settings. The aim of this study was to quantify the burden and determine predictors of non-urgent surgical ER visits in a tertiary care setting.

Methods: This is a prospective analysis of the non-urgent visits in our SER over a 15-day period. We included patients of all ages and genders who presented to the SER at the time of data collection. Patients were excluded if they were detained, dead on arrival, or refused to consent. Patients were stratified into two groups based on the surgical urgency: group U (urgent) and group NU (non-urgent), and compared for the baseline characteristics, comorbid conditions, and referral route. Multivariable analyses were used to identify the independent predictors of these non-urgent presentations while controlling for potential confounders.

Results: Non-urgent visits accounted for 147 (35%) of all visits and were associated with younger age, female sex, rural residence, low income, and limited education (p<0.001). Most non-urgent cases were triage-referred (110 (74.8%), p=0.012). Diabetes mellitus (DM) and obesity were more common in urgent cases (p<0.05). Independent predictors of non-urgent visits included female sex (adjusted odds ratio (aOR): 1.50, 95% CI (1.10 - 2.05), p = 0.020), rural residence (aOR: 1.30, 95% CI (1.02 - 1.65), p < 0.040), low income (aOR: 1.40, 95% CI (1.05 - 1.88), p = 0.030), and chronic kidney disease (CKD) (aOR: 1.80, 95% CI (1.10 - 3.20), p = 0.040).

Conclusion: Our study identified the common predictors of non-urgent SER visits in our settings, such as younger age, rural residence, poor socioeconomic status, and lower education level. Combined efforts should focus on improving the triage protocols, access to primary care, patient education, and public health initiatives. Further research should also assess the effectiveness of urgent care clinics and telemedicine in reducing non-urgent ER presentations.

## Introduction

With the rapidly advancing medical sciences and the introduction of subspecialty-based care, appropriate triaging has become necessary to ensure patient care and balance the workload of healthcare workers [1]. Like other departments, surgical teams contribute their share in managing patients with surgical complaints [2]. However, it is a common and frequent observation that not all the patients presenting to the surgical emergency room (SER) have an indication to be seen there [3]. Most of them are non-urgent presentations, while others have emergency medical, psychiatric, obstetric, or other presentations that are better treated elsewhere. This leads to overcrowding in the SER, misallocation of resources, longer waiting times, and potential delays in the management of true surgical emergencies [4]. This, in turn, is associated with increased morbidity, prolonged hospital stays, and higher mortality rates [4]. Differentiating between urgent and non-urgent surgical presentations is essential for effective triage, resource allocation, and patient management [5].

While the problem of this inappropriate burden of non-surgical cases in the SERs is prevalent around the globe, the repercussions are expected to be particularly concerning in developing countries [6]. Hospitals in these countries have inadequate resources to accommodate even the baseline patient volumes [7]. Previous studies have shown that several patient-related factors, including lack of health literacy, poor socioeconomic status, and lack of access to primary care services, significantly affect the inappropriate use of emergency services [8]. Other factors may include the apprehension of prolonged waiting times in outpatient clinics and financial costs. Addressing these issues requires targeted health education programs and policy interventions to enhance the efficiency of emergency surgical care [9].

This study aimed to assess the frequency and predictors of non-emergency and non-urgent visits to the SER of a tertiary care hospital in Peshawar, Pakistan. Our hypothesis was that a significant proportion of patients presenting to our SER do not have an appropriate indication for urgent surgical evaluation. This study will allow us to quantify the magnitude of this issue, identify key factors contributing to inappropriate SER utilization in this population, and provide evidence-based recommendations for improving triage protocols and patient education.

## Materials and methods

Study design and settings

This prospective, comparative, cross-sectional study was conducted over a 15-day period (9^th^ January to 23^rd^ January 2023) in the surgical emergency department of Hayatabad Medical Complex, a major teaching hospital in Peshawar, Pakistan.

Data collection and sampling technique

After approval from the Institutional Review Board (IRB) of Hayatabad Medical Complex (approval number: 1021) and informed consent from the patients or their families, we enrolled them in our study using a modified convenience sampling technique. The 24-hour day was divided into four six-hour blocks, selecting one block of observation per day for patient enrollment. This approach ensured balanced representation across different shifts while maintaining feasibility in the busy emergency setting.

Inclusion and exclusion criteria

We included all patients presenting to the SER part of the Accident and Emergency (A&E) department of the hospital. Patients were excluded if they didn't consent to participate in the study. We also excluded detained individuals to maintain focus on the target population and ensure ethical compliance.

Sample size calculation

Considering an anticipated prevalence of 61% of non-urgent visits to the emergency department [5], we estimated a sample size of 366 patients, with a 95% confidence level, 5% margin of error, and a desired power of 80% using the standard proportion formula. Anticipating a dropout rate of 10% to 12% due to potential incomplete medical records and patients leaving before complete evaluation, we collected data from a total of 420 patients. The sample size calculation was carried out using the free OpenEpi software (www.OpenEpi.com)[10].

Study population and stratification

Patients who presented to the SER were categorized as Group U (urgent cases that required immediate or emergency surgical evaluation with or without intervention) and Group NU (non-urgent cases, including those that could have been managed electively and those with non-surgical emergencies). These were identified by physician assessment.

Data points

Using a detailed proforma (Appendix A), we collected data on demographic characteristics (age, gender, and residence location), socioeconomic indicators (education level and income category), comorbid conditions (diabetes mellitus (DM), hypertension (HTN), chronic kidney disease (CKD), and congestive heart failure (CHF)), chief complaints, symptom duration, and referral pathway. We also collected data on the outcome measures focused on key performance indicators, including management pathways (observation, admission, or discharge), discharge disposition, and mortality. The proforma was specifically designed by the research team for this particular study. Using a pilot study of 15 patients, the proforma was modified to remove ambiguities, improve clarity, and ensure completeness of the data collected. 

Statistical analyses

All statistical analyses were performed using the IBM SPSS Statistics software, version 23 (IBM Corp., Armonk, NY). Continuous variables were reported as mean ± standard deviation (SD). The two groups were compared for means of normally distributed continuous baseline variables using independent sample t-tests, while non-normal data were compared with Mann-Whitney U tests and presented as median (interquartile range). Categorical variables, including our primary outcomes, were expressed as frequencies and percentages and analyzed using Pearson's chi-square or Fisher's exact tests, as indicated. To identify the independent association of different baseline and comorbid conditions, we conducted the multivariable regression analyses adjusting for potential confounders. Model fit was assessed using the Hosmer-Lemeshow goodness-of-fit test. A p <0.05 was considered statistically significant for all analyses.

## Results

Baseline characteristics

A total of 420 patients were enrolled in this study, of whom 147 (35.0%) presented with non-urgent complaints and 273 (65.0%) were categorized as urgent presentations. The mean ± SD age of the cohort was 42.9 ± 16.0 years, with group NU being significantly younger than group U (38.7 ± 14.2 vs. 45.2 ± 16.8 years, p < 0.001). Women constituted 204 (48.6%) of the total sample, and their proportion was higher in the non-urgent group compared to the urgent group (p=0.003) (Table 1).

**Table 1:** Comparison of Baseline Characteristics of Patients Presenting with Urgent vs. Non-urgent Complaints to the Surgical Emergency Room SD: standard deviation; low income <20K Pakistani Rupees (PKR)/month Continuous Variables Are Reported As Mean ± SD Unless Otherwise Specified. Categorical Variables Reported As Frequency (Percentage); ‡ Fisher’s Exact Test Used for Categorical Variables With Expected Cell Counts <5. P-values Are Calculated Using Two-Tailed Tests With α=0.05; Significant Results (p<0.05) Are Highlighted in Bold.

Variable	Total Cases (n=420)	Non-urgent Visits (n=147)	Urgent Visits (n=273)	P-value	Test Statistic (Test Used)
Age (years), Mean±SD	42.9 ± 16.0	38.7 ± 14.2	45.2 ± 16.8	<0.001	-3.99 (χ²)
Female Sex	204 (48.6%)	86 (58.5%)	118 (43.2%)	0.003	8.33 (χ²)
Rural Residence	154 (36.7%)	70 (47.6%)	84 (30.8%)	<0.001	10.97 (χ²)
Low Income ^a^	168 (40.0%)	77 (52.4%)	91 (33.3%)	<0.001	13.66 (χ²)
Poor Socioeconomic Status	182 (43.3%)	84 (57.1%)	98 (35.9%)	<0.001	14.22 (χ²)
Education Levels (≤Primary)	126 (30.0%)	62 (42.2%)	64 (23.4%)	<0.001	16.31 (χ²)
Triage Referral	280 (66.7%)	110 (74.8%)	170 (62.3%)	0.012	6.30 (χ²)
OPD/Clinic Referral	140 (33.3%)	37 (25.2%)	103 (37.7%)	<0.001	6.78 (χ²)

Socioeconomic disparities were quite obvious between groups. A significantly higher proportion of non-urgent cases were from rural areas (74 (47.6%) vs. 80 (30.8%), p=0.001), had low income (77 (52.4%) vs. 91 (33.3%), p<0.001), and belonged to lower socioeconomic status (84 (57.1%) vs. 98 (35.9%), p<0.001). Additionally, group NU frequently had a lower education level (62 (42.2%) vs. 64 (23.4%), p <0.001) (Table 1).

Referral patterns

Notably, 110 (74%) of non-urgent cases originated from triage referrals compared to 170 (62%) of urgent cases (p < 0.05). In contrast, OPD/clinic referrals were more likely to be urgent (37 (25%) vs. 103 (37%), p < 0.001), indicating better pre-selection in structured outpatient settings (Table 1).

Comorbid conditions

Several comorbid conditions were more common in the group NU, of which the prevalence of CKD was significantly high in this group compared to group U (18 (5.8%) vs. four (2.3%), p=0.048), while differences in other comorbidities did not reach statistical significance, including HTN (92 (29.7%) vs. 42 (23.9%), p=0.093). heart disease (55 (17.7%) vs. 23 (13.1%), p=0.194) and chronic liver disease (31 (10.0%) vs. 17 (9.7%), p=0.940), chronic obstructive pulmonary disease (COPD)/asthma (41 (13.2%) vs. 16 (9.1%), p=0.243), or stroke history (21 (6.8%) vs. 8 (4.5%), p=0.404). In contrast, group U was more frequently diabetic (25 (17%) vs. 87 (31.9%), p=0.003) and obese compared to group NU (22 (15%) vs. 67 (24.5%), p=0.014) (Table 2).

**Table 2:** Comparison of Comorbid Conditions of Patients Presenting with Urgent vs. Non-urgent Complaints to the Surgical Emergency Room COPD: Chronic Obstructive Pulmonary Disease; CAD: Coronary Artery Disease; CHF: Congestive Heart Failure Categorical Variables Reported As Frequency (Percentage); ‡ Fisher’s Exact Test Used for Categorical Variables With Expected Cell Counts <5; P-values Are Calculated Using Two-Tailed Tests With α=0.05; Significant Results (p<0.05) Are Highlighted in Bold.

Variable, n(%)	Total Cases (n=420)	Non-urgent Visits (n=147)	Urgent Visits (n=273)	P-value	Test Statistic (Test Used)
Diabetes Mellitus	112 (22.4%)	25 (17.0%)	87 (31.9%)	0.003	8.600 (χ²)
Obesity	89 (17.8%)	22 (15.0%)	67 (24.5%)	0.014	2.740 (χ²)
Hypertension	134 (26.8%)	92 (29.7%)	42 (23.9%)	0.093	2.800 (χ²)
Active Malignancy	15 (3.0%)	10 (3.2%)	5 (2.8%)	0.978	0.001 (Fisher’s Exact)
Chronic Liver Disease	48 (9.6%)	31 (10.0%)	17 (9.7%)	0.940	0.005 (χ²)
Chronic Kidney Disease	22 (4.4%)	18 (5.8%)	4 (2.3%)	0.048	3.900 (Fisher’s Exact)
COPD/Asthma	57 (11.4%)	41 (13.2%)	16 (9.1%)	0.243	1.360 (χ²)
Heart Disease (CAD/CHF)	78 (15.6%)	55 (17.7%)	23 (13.1%)	0.194	1.690 (χ²)
Previous Stroke	29 (5.8%)	21 (6.8%)	8 (4.5%)	0.404	0.690 (Fisher’s Exact)

Independent predictors of non-urgent presentation

Multivariable regression analysis identified several independent predictors of non-urgent emergency department visits. Younger age was associated with increased odds of non-urgent presentation (adjusted odds ratio (aOR): 1.01, 95% CI (1.00-1.02), p=0.052). Female sex (aOR: 1.50, 95% CI (1.10-2.05), p=0.020) and rural residence (aOR: 1.30, 95% CI (1.02-1.65), p=0.040) were also significant predictors.

Lower socioeconomic status remained an independent factor. Patients with low income had 1.4 times higher odds of presenting with non-urgent complaints (aOR: 1.40, 95% CI (1.05-1.88), p=0.030), and those with lower education had 1.35 times higher odds (aOR: 1.35, 95% CI (1.00-1.80), p=0.050).

Among comorbidities, hypertension (aOR: 1.50, 95% CI (1.05-2.10), p=0.030), CKD (aOR: 1.80, 95% CI (1.10-3.20), p=0.040), heart disease (aOR: 1.45, 95% CI (1.05-2.00), p=0.035), and chronic liver disease (aOR: 1.60, 95% CI (1.02-2.55), p=0.045) were significantly associated with non-urgent presentation. The model demonstrated good fit, as confirmed by the Hosmer-Lemeshow test (p=0.51) (Table 3).

**Table 3:** Multivariable Regression Analysis for the Independent Association of Baseline Characteristics and Comorbid Conditions on Non-urgent Presentation CKD: Chronic Kidney Disease, aOR: Adjusted Odds Ratio, CI: Confidence Interval The Hosmer-Lemeshow Test Confirms Good Model Fit (P = 0.51). Significant Results (p<0.05) Are Highlighted in Bold.

Predictors	aOR	95% CI	P-value
Young Age	1.01	1.00 – 1.02	0.052
Female	1.50	1.10 – 2.05	0.020
Rural Residence	1.30	1.02 – 1.65	0.040
Low Income	1.40	1.05 – 1.88	0.030
Low Education	1.35	1.00 – 1.80	0.050
Hypertension	1.50	1.05 – 2.10	0.030
CKD	1.80	1.10 – 3.20	0.040
Heart Disease	1.45	1.05 – 2.00	0.035
Chronic Liver Disease	1.60	1.02 – 2.55	0.045

## Discussion

Emergency health services are the cornerstone of any healthcare system, but their inappropriate use can severely compromise their effectiveness and patient outcomes [11]. In this study, we observed that patients presenting to the SER with non-urgent conditions were typically younger, more frequently female, and resided in rural areas. We also found that poor socioeconomic status and lower educational levels were independently associated with an increased likelihood of these non-urgent visits. While these patients received comprehensive evaluations as necessary, the high proportion of these non-urgent presentations adversely affected the overall patient care. Though establishing an ideal emergency care system might require years to decades, studies such as ours provide critical insights and evidence for continuous improvement.

While most adults adequately tolerate their symptoms, younger adults seek out urgent attention for even minor complaints due to anxiety and unawareness. O’Keeffe et al. reported a high rate of non-urgent presentation in younger patients, similar to our study [12]. The age curve in this group might also be more skewed due to the pediatric patients in our sample, as parents of young children tend to get concerned when their little ones are sick. In terms of gender distribution, non-urgent visits were more common among female patients (58%). Alnasser et al. also reported a higher proportion of females among their non-urgent group (63%) [5]. Firstly, we may assume that male patients tend to ignore their symptoms, while females are more health-conscious and tend to seek prompt treatment. Another factor is the predisposition of the female gender to somatization of symptoms that bring patients to the ER with complaints that appear to be emergencies to the patients, but on evaluation, they don’t have any emergency indications and are thus mostly discharged home without any need for advanced care [13].

Lower socioeconomic status and limited education were key predictors of non-urgent SER visits, a connection likely to arise from reduced health awareness and limited access to appropriate healthcare services among these groups [5,8]. Some recent studies have highlighted patient perception of illness severity, but individuals with lower health literacy also tend to perceive minor symptoms as serious emergencies [1]. In addition, emergency departments typically provide around-the-clock care and a wider range of services under one roof [2], adding an element of trust and convenience to patients' choices. Qualitative research on patient perceptions, motivations, and decision-making would provide invaluable insights, allowing healthcare planners to better design interventions that reduce inappropriate ER utilization and enhance overall patient care.

While international studies often attribute the high frequency of non-urgent ER visits to the excessive costs of outpatient care [9], this explanation does not apply to our setting, since basic outpatient services here remain affordable in public hospitals like ours. However, quicker availability of laboratory tests and diagnostic procedures in the emergency department may encourage patients to prefer ER visits over outpatient clinics [3]. Similar patterns have been reported in other welfare countries like Canada and the United Kingdom, where emergency departments provide rapid diagnostics compared to the long waiting times in outpatient consultations, sometimes as long as months or even years [6, 4]. Because our study was limited to the ER only, we could not directly assess whether differences in diagnostic waiting times influence patient decisions. Further studies should compare the diagnostic timelines between emergency and outpatient departments and provide guidelines on how to expedite our outpatient services.

Primary care providers (PCPs) play a vital role in identifying and directing surgical emergencies [9, 14]. Recent evidence emphasizes that inadequate primary care infrastructure significantly contributes to excessive emergency department use, especially in our setting, where the concept of designated PCPs is almost nonexistent [7, 5, 15]. In the absence of an assigned PCP, most of our referrals come from other generalists, specialists, or self-referrals that are triaged in the ER. Most of our non-urgent surgical visits to SER were referred by triage nurses and casualty medical officers, whereas genuine emergencies are typically identified by primary care or outpatient physicians, aligning with results from other studies [9, 3]. In addition to improving our triage training, our general focus should also include strengthening our primary care services and educating patients about appropriate emergency department use.

Some comorbid conditions, including CKD, HTN, and heart disease, were more commonly seen with non-urgent surgical visits, consistent with other research [16, 5]. A likely explanation in our population can be the incorrect triage of patients with chronic medical conditions into surgical emergency rooms instead of medical units. Patients with chronic illnesses often disproportionately rely on emergency departments due to challenges in accessing routine healthcare [8]. In contrast, diabetes and obesity were more prevalent among genuinely urgent surgical cases, likely reflecting the association of these conditions with diabetic foot ulcers, abscesses, carbuncles, and necrotizing infections, all of which require urgent surgical intervention [17, 18].

Limitations

This study might have some important limitations to mention. Some patients, especially critically ill patients who need urgent transfer to the OR, ICU, or another facility, might have been missed in the busy SER, leading to selection bias. The cross-sectional design only collected data on the ER visit itself without follow-up on patient outcomes outside the ER. In addition, the overlap of symptoms of some medical and surgical conditions may have caused errors in the categorization of certain conditions. Lastly, this was a single-center, small-sample study in the SER only, which may limit its generalizability. Future studies with larger, multicenter designs and longitudinal follow-up are needed to confirm our findings and explore interventions for reducing non-urgent ED utilization.

## Conclusions

Our study identified the common predictors of non-urgent SER visits in our settings, such as younger age, female sex, rural residence, lower socioeconomic status, lower education levels, and chronic medical conditions, which add an unnecessary burden on emergency care. Combined efforts from clinicians, ER physicians, and the government should focus on improving the triage protocols, better access to primary care, patient education, and public health initiatives. Future research should also assess the effectiveness of alternative care settings, like urgent care clinics and telemedicine, in reducing non-urgent ED presentations.

## Appendices

Appendix A 

**Table 4:** Proforma for Data Collection PKR: Pakistani Rupees

Title: Burden and Predictors of Non-Urgent Visits in the Surgical Emergency Room. The purpose of these data is to assess the prevalence of non-urgent and non-surgical emergency visits in the surgical emergency room of Hayatabad Medical Complex, Peshawar, Pakistan. These data will remain confidential and anonymous and will be retained only for research purposes.		
Date		
Assigned Serial Number	Age	Gender
Education level:	Monthly Income in PKR:	Address of Residence:
Referred by:	Presenting Complaints	Diagnosis:
Comorbid Conditions: (specify)	Urgency Status:	Management and Discharge Disposition
